# Nanoformulation-Based Antiviral Combination Therapy for Treatment of COVID-19

**Published:** 2020

**Authors:** Hoda Keshmiri Neghab, Seyedeh Sara Azadeh, Mohammad Hasan Soheilifar, Fariba Dashtestani

**Affiliations:** 1. Department of Photo Healing and Regeneration, Medical Laser Research Center, Yara Institute, ACECR, Tehran, Iran; 2. Department of Biology, Science and Research branch, Islamic Azad University, Tehran, Iran; 3. Department of Medical Laser, Medical Laser Research Center, Yara Institute, ACECR, Tehran, Iran; 4. Institute of Biochemistry and Biophysics, University of Tehran, Tehran, Iran

In December 2019, a novel coronavirus disease (COVID-19) was detected in Wuhan, China, which was accompanied by symptoms of fever, dry cough, weakness of immune system with reduction of the white blood cells. Afterwards, coronavirus has speared and affected many countries in worldwide ^1,2^.

Coronaviruses are subgroup of enveloped, single-stranded, positive-sense RNA genome, phylogenetically associated with acute respiratory syndrome coronavirus (SARS-CoV-2) and Middle East Respiratory Syndrome Coronavirus (MERS-CoV) which are closely related to lung disease leading to Acute Respiratory Distress Syndrome (ARDS) ^3^.

It has been shown that coronavirus spike protein (S protein) is a transmembrane glycoprotein responsible for cell entry into the target cells. It binds to the host cell receptors, Angiotensin Converting Enzyme 2 (ACE-2), inducing conformational rearrangement that drives membrane fusion ^4^. Since the primary goal of antiviral therapy is blocking virus entry, inhibition of ACE-2 as a main entry key into cells for coronavirus is the most promising therapeutic approach ^4^. Although ACE-2 is widely distributed in the human body, and can affect a variety of organs and cells including the lung, heart, kidney, and small intestine, but promising and effective potential of ACE-2 in blocking viral entry, inhibiting inflammation and reducing the damage of target organs cannot be ignored even after some adverse effects ^4^. DX600 is a novel, specific and potent peptide inhibitor of ACE2 that can be used in antiviral treatment for covid-19 ^5^.

In addition to ACE-2 blocking strategy, preventing virus replication by inhibition of host inosine monophosphate dehydrogenase (IMPDH) enzyme as a crucial goal of antiviral therapy for virus infection could be a potential strategy in the battle against coronavirus. Ribavirin is analogue of purine nucleoside that can inhibit the IMPDH enzyme to prevent replication of the genetic material (RNA and DNA) of viruses ^6^. It has been revealed that taking Ribavirin by both intravenous and oral administration at different doses is the effective way for prevention and treatment of SARS ^7^. But taking medication at high doses leads to adverse effects such as nausea, exacerbation of bronchospasm and dose-dependent anemia; even it may affect the embryo and cause a genetic mutation ^7^.

It has been reported that Combination Antiretroviral Therapy (CART) promotes the effectiveness of the treatment and decreases the risk of drug resistance ^8^. Hence, administration of Ribavirin and DX600 which are two antiviral agents would be more effective.

Moreover, nanomedicine with its rapid growth combines the nanotechnology with the biomedical and pharmaceutical sciences ^9^. The small particle size of Nanoparticles (NPs) creates dispersity in the stable nanostructure and enables easy entrance into cells; thereby, drug cost is reduced and the therapeutic efficacy will improve. Moreover, the dose of drug to be administered can be reduced and concurrently the unwanted side effects are minimized.

**Figure 1.**
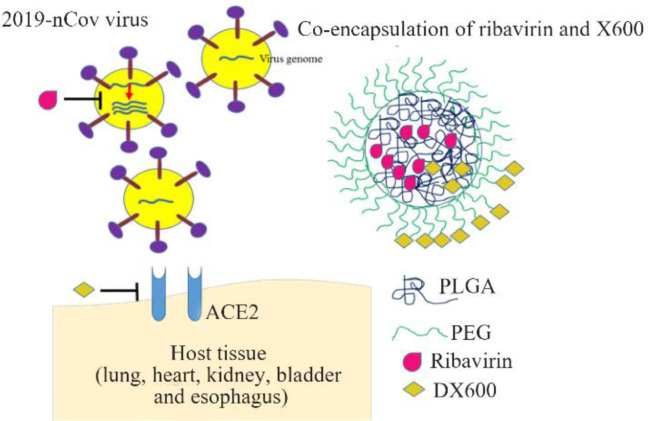
Schematic structure of polymeric nanosystem platform for antiviral combination therapy of COVID-19.

So, by including Nanoparticles (NPs) in drug formulations, the efficacy, safety, and dose of administered drug would be improved. The formidable barriers for gastrointestinal tract, skin and cell have limited the therapeutic effects of antiviral drugs. For example, functionalized single-walled carbon nanotubes were used as a nanodrug carrier for Ribavirin for the treatment of viral diseases in fish. The results show that Ribavirin intake was increased by nanocarrier and therapeutic dosage was significantly reduced ^10^. Several researches have been conducted about antiviral drug delivery nanosystems and their transport across specific barriers at cellular and intracellular level ^11^. Furthermore, improving the antiretroviral agents' delivery could overcome some probable limitations of current CART ^12^. Thus, co-encapsulation of Ribavirin and DX600 in NPs would afford a novel means of drug delivery to SARS-CoV-2 in tissues. The studies in mice and in human cell lines revealed that DX600 is a potent ACE2 inhibitor specific for only human ACE2 ^13^. Hence, by using DX600 in nano-formulation, the targeted drug delivery would be obtained. Polylactic-co-glycolic acid (PLGA) and polyethylene glycol (PEG) copolymer is a developed system with many advantages; it is biodegradable, biocompatible, easily synthesized by self-assembling into nanometric micelles, and it favors a time-dependent release manner, reduces blood clearance of nanocarriers and increases blood circulation time ^14^. Consequently, co-encapsulation of Ribavirin and DX-600 in PLGA-PEG provides access to all good qualities of antiviral drugs and nanocarriers in one drug. Schematic representation of using nanotechnology systems for delivery of antiretroviral drugs has been shown in figure 1.

In the end, it is hypothesized that the use of PLGA-PEG copolymer to deliver two or more antiretroviral drugs to suppress viral entry and viral replication by DX600 and Ribavirin, respectively can be a promising tool in treatment of coronavirus. Moreover, PLGA-PEG copolymer has the potential to be applied as a nanocarrier agent for codelivery of any antiviral drugs to target tissue and consequently promotes the CART in living organisms.
